# Evans Syndrome in a 35-Year-Old Male: Diagnostic Challenges and Multimodal Management

**DOI:** 10.7759/cureus.92042

**Published:** 2025-09-11

**Authors:** Anjiya Aswani, Domonick K Gordon, Syed Sajjad Badshah, Mohamed Moctar, Omar Kayyas

**Affiliations:** 1 Internal Medicine, Ross University School of Medicine, Bridgetown, USA; 2 Hematology and Oncology, Mount Sinai Hospital, Chicago, USA

**Keywords:** autoimmune haemolytic anemia, corticosteroids, direct antiglobulin test, evans’ syndrome, immune thrombocytopenia (itp), rare diseases, rituximab, thrombocytopenia

## Abstract

Evans syndrome (ES) is a rare autoimmune disorder characterized by the co-occurrence or sequential development of autoimmune hemolytic anemia (AIHA) and immune thrombocytopenia (ITP), and less frequently, neutropenia. It is a diagnosis of exclusion that can be challenging to recognize due to its nonspecific presentation and overlap with other hematologic or autoimmune disorders. We report the case of a 35-year-old previously healthy male who presented with generalized weakness and extensive bruising. He was hemodynamically stable on admission but exhibited diffuse petechiae and ecchymoses without evidence of hepatosplenomegaly or lymphadenopathy. Laboratory evaluation revealed severe normocytic anemia and profound thrombocytopenia, along with biochemical markers of hemolysis. The direct antiglobulin test (DAT) was positive for IgG, consistent with warm AIHA. The peripheral smear showed marked anisopoikilocytosis, slight schistocytosis, moderate hypochromasia, microspherocytes, and a markedly reduced platelet count. A comprehensive workup, including autoimmune and infectious panels, was unremarkable. Bone marrow biopsy demonstrated erythroid hyperplasia, increased immature megakaryocytes, and no evidence of malignancy, supporting a diagnosis of ES. The patient required multiple transfusions and was treated with high-dose corticosteroids, intravenous immunoglobulin (IVIG), and rituximab, with gradual improvement in cell counts. Second-line agents, such as eltrombopag, represent important therapeutic options in refractory cases, although they were not required for this patient. This case highlights the importance of maintaining a high index of suspicion for ES in patients presenting with unexplained cytopenias, particularly when evidence of hemolysis is present. Early identification and prompt initiation of immunosuppressive therapy can improve outcomes in this potentially life-threatening condition.

## Introduction

Evans syndrome (ES) is a rare autoimmune disorder defined by the simultaneous or sequential occurrence of autoimmune hemolytic anemia (AIHA) and immune thrombocytopenia (ITP), with or without immune neutropenia [1,2]. The pathophysiology is complex and not fully understood, but is thought to involve immune dysregulation, including polyclonal B-cell activation, autoantibody production, and impaired regulatory T-cell function [1,3,4]. ES may occur as a primary idiopathic condition or secondary to other diseases such as systemic lupus erythematosus, autoimmune lymphoproliferative syndrome, common variable immunodeficiency, lymphomas, or infections [2,5-7]. Genetic susceptibility and environmental factors may also contribute, although the evidence remains limited [2-4,8].

Risk factors for developing ES include a personal or family history of autoimmune disease, underlying immunodeficiency, and prior lymphoproliferative disorders [2,5-7]. Infectious triggers have also been implicated, particularly viral illnesses that can precipitate or exacerbate autoimmune cytopenias [4,8]. These factors highlight the multifactorial nature of ES and the importance of recognizing potential associations to guide both diagnosis and long-term management.

Clinically, ES presents with variable severity, ranging from mild cytopenias to life-threatening bleeding, hemolysis, or thrombotic complications [1,2,9]. Diagnosis requires a high index of suspicion, particularly in patients with unexplained recurrent cytopenias or poor response to standard therapies for isolated AIHA or ITP [5,10]. Management often begins with corticosteroids or intravenous immunoglobulin (IVIG), but ES frequently follows a relapsing-remitting course and is more refractory to treatment compared with isolated cytopenias, necessitating second-line options such as rituximab, immunosuppressive agents, or thrombopoietin receptor agonists [6,9,11-13].

We present a case of idiopathic ES that was refractory to steroids and IVIG but treated with rituximab and eltrombopag, highlighting a multimodal approach to early second-line therapy and the importance of nutritional support in patients with comorbid risk factors.

## Case presentation

A 35-year-old male with a history of alcohol use disorder and prediabetes presented with a two-week history of fatigue, exertional dyspnea, petechiae, and widespread ecchymoses. He initially noticed spontaneous bruising on the abdomen, which gradually progressed to other body areas. He also reported intermittent blood-streaked sputum and hematochezia, both of which resolved before admission. He denied hematuria, melena, epistaxis, recent infections, or known autoimmune or hematologic disorders. On the day of admission, he experienced dizziness and near-syncope while showering.

On physical examination, the patient was afebrile and hemodynamically stable. Petechiae and ecchymoses were observed over the trunk and extremities. There was no scleral icterus, lymphadenopathy, or hepatosplenomegaly. Laboratory studies revealed severe anemia and thrombocytopenia, with additional findings consistent with hemolysis and elevated reticulocyte count, as summarized in Table 1.

**Table 1:** Hematologic laboratory results at presentation. Laboratory reference ranges are based on institutional standards at Mount Sinai Hospital, Chicago, which may vary slightly between centers. *Elevated LDH (669 U/L; reference 140-280 U/L) was consistent with active hemolysis. All values are reported in conventional units.

Laboratory test	Result	Reference range
Hemoglobin	5.7 g/dL	13.5-17.5 g/dL (male)
Platelet count	7 × 10⁹/L	150-400 × 10⁹/L
White blood cell count	9.7 × 10⁹/L	4.5-11.0 × 10⁹/L
Reticulocyte count	13.90%	0.5%-2.5%
Lactate dehydrogenase (LDH)	669 U/L	140-280 U/L*
Total bilirubin	1.6 mg/dL	0.1-1.2 mg/dL
Haptoglobin	101 mg/dL	30-200 mg/dL
Direct antiglobulin test	Positive for IgG only	Negative
Partial thromboplastin time (PTT)	25.2 seconds	25-36 seconds
Prothrombin time (PT)	12.3 seconds	10.1-13.1 seconds
International normalized ratio (INR)	1.1	0.9-1.2

**Figure 1:**
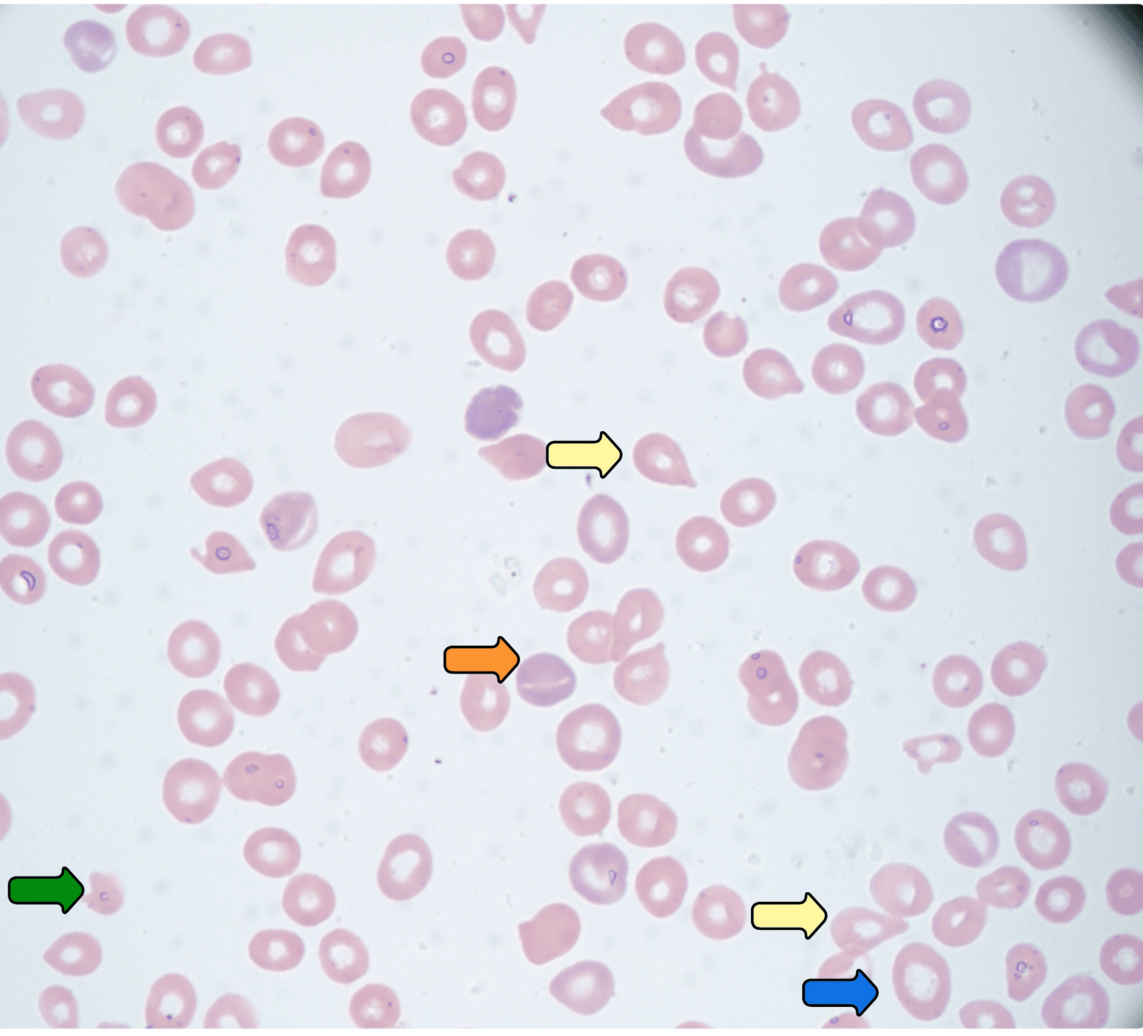
Peripheral blood smear demonstrating marked anisopoikilocytosis. Representative findings are indicated: teardrop cells (yellow arrows), polychromasia (orange arrow), macrocytes (blue arrow), and a schistocyte (green arrow).

**Figure 2:**
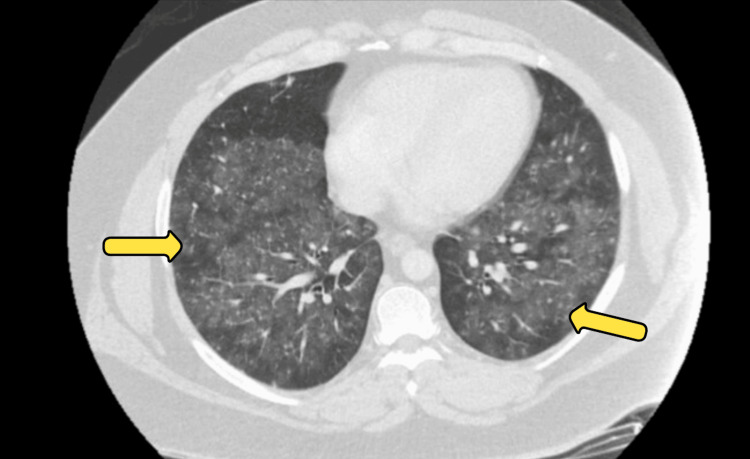
Axial CT chest demonstrating multifocal bilateral ground-glass opacities (arrows).

The peripheral smear revealed marked anisopoikilocytosis, with macrocytes, microcytes, moderate hypochromasia, polychromasia, teardrop cells, elliptocytes, microspherocytes, slight schistocytes, and acanthocytes (Figure 1). Occasional nucleated red blood cells and rare blasts were observed, along with significantly reduced platelets. Serum protein electrophoresis showed a faint IgM kappa spike, but the kappa/lambda ratio remained within normal limits (0.94). Given the presence of rare blasts and a paraprotein band, additional evaluation was pursued to exclude an underlying clonal process. Autoimmune and infectious workup, including antinuclear antibody (ANA), breakpoint cluster region-Abelson (BCR-ABL) testing, HIV, and hepatitis panels, was negative. Coagulation studies, including prothrombin time (PT), partial thromboplastin time (PTT), and international normalized ratio (INR), were within normal limits, helping to rule out a consumptive coagulopathy such as disseminated intravascular coagulation (DIC). Flow cytometry revealed no evidence of clonal hematologic malignancy. CT imaging of the chest was unremarkable for lymphadenopathy or masses (Figure 2) but demonstrated multifocal bilateral ground-glass opacities, suggestive of pneumonia. Based on these findings, the patient was started on ceftriaxone and azithromycin with a good clinical response. Bone marrow biopsy demonstrated a hypercellular marrow with erythroid hyperplasia, increased immature megakaryocytes, left-shifted granulopoiesis, and mild eosinophilia, consistent with immune-mediated hematologic destruction.

Initial treatment included transfusions of packed red blood cells (PRBCs) to maintain hemoglobin above 6 g/dL. Platelet transfusions were minimally effective. High-dose intravenous methylprednisolone (1 mg/kg) was started on hospital day one, followed by intravenous immunoglobulin (IVIG, 1 g/kg/day) for two days, beginning on hospital day 2. Rituximab infusion (1,000 mg in 1,000 mL NS) was added on hospital day 4 due to persistent thrombocytopenia. Nutritional support with folic acid, thiamine, multivitamins, and intravenous iron was initiated. Allopurinol was prescribed for hyperuricemia. Despite therapy, lactate dehydrogenase (LDH) and reticulocyte counts remained elevated with minimal platelet recovery. Bone marrow biopsy findings confirmed the clinical suspicion of ES.

During hospitalization, the patient developed community-acquired pneumonia, which was successfully treated with ceftriaxone and azithromycin. He remained hemodynamically stable throughout his stay. At discharge, he was prescribed oral prednisone (100 mg daily) and eltrombopag (50 mg daily), with plans for outpatient rituximab infusion and hematology follow-up. He was instructed to seek urgent medical care for any signs of bleeding or clinical deterioration.

## Discussion

ES remains a diagnostic and therapeutic challenge due to its rarity, heterogeneity, and often relapsing course [1,3]. The patient’s initial presentation with severe anemia and profound thrombocytopenia, along with positive direct antiglobulin testing and absence of an underlying secondary cause, was consistent with idiopathic ES [4,5]. Bone marrow biopsy findings of erythroid hyperplasia and increased immature megakaryocytes further supported peripheral destruction rather than primary marrow failure [2,6].

First-line therapy for ES typically includes high-dose corticosteroids and/or IVIG [4,7]. However, responses are often incomplete or transient, and up to 60% of patients require second-line agents [3,8]. In this case, the patient’s lack of platelet response prompted the early initiation of rituximab, a monoclonal anti-CD20 antibody that has demonstrated efficacy in refractory ES and is now considered a standard second-line option [5,9,10]. The addition of eltrombopag reflects emerging evidence supporting thrombopoietin receptor agonists for persistent thrombocytopenia in ES, including limited reports of use in the acute phase [1,2,8]. While most data involve chronic or refractory cases, small case series and reports suggest eltrombopag can rapidly increase platelet counts in acute settings when standard therapy is insufficient [2,3,9,14]. 

Nutritional support was also vital, given the patient’s alcohol use and elevated reticulocyte count, suggesting active erythropoiesis. Empiric vitamin supplementation, including folic acid and thiamine, along with IV iron, is frequently indicated in such patients to support hematologic recovery [11,12]. The patient’s interim development of community-acquired pneumonia, occurring after the CT chest investigation, also underscores the infection risk associated with immunosuppressive therapy in ES [1,4].

Though rare, ES is increasingly recognized due to enhanced awareness and consensus definitions. The 2024 international consensus guidelines provided a more precise definition of ES, requiring the presence of at least two autoimmune cytopenias (most commonly AIHA and ITP, with or without immune neutropenia) after exclusion of secondary causes [1]. These updated definitions improve diagnostic consistency and facilitate earlier recognition. This case reinforces the need for early suspicion, comprehensive evaluation, and a multidisciplinary approach for optimal management of ES. In addition to hematology, involvement of infectious disease specialists helps mitigate infectious risks, while nutrition and internal medicine support are essential for addressing comorbidities and promoting recovery [11,13]. Such collaboration enables individualized care planning, particularly for patients with refractory or relapsed disease. 

Despite recent advancements, the optimal management of ES remains unclear due to a lack of large-scale, prospective trials. Further research is needed to better define the disease’s underlying immunopathogenesis, establish standardized diagnostic criteria, and evaluate the long-term safety and efficacy of emerging therapies such as thrombopoietin receptor agonists and biologics. Collaborative registries and multicenter studies may help generate evidence to guide personalized treatment strategies and improve outcomes in this rare and complex disorder.

## Conclusions

ES is a rare and diagnostically challenging autoimmune disorder characterized by the coexistence of autoimmune hemolytic anemia and immune thrombocytopenia. Management often requires aggressive and multimodal immunosuppressive therapy, yet relapses are frequent, highlighting the importance of individualized treatment approaches. This case emphasizes the need to consider ES in patients presenting with refractory cytopenias and evidence of hemolysis after other potential causes have been ruled out. Moving forward, prospective studies and multicenter registries are needed to evaluate the safety and efficacy of emerging therapies, define standardized diagnostic criteria, and identify predictors of treatment response. Such efforts will inform evidence-based clinical strategies and improve outcomes for patients with this complex disorder.
